# Rupture of the Œsophagus

**Published:** 1900-08-11

**Authors:** 


### RUPTURE OF THE ŒSOPHAGUS.

Considering the frequency with which people vomit, and the rarity of any evil effect upon the œsophagus from so doing, there can be but little doubt that when such a disaster as rupture of the œsophagus occurs something more than mere vomiting or retching has been at work in its production—some preceding injury or disease by which the strength and elasticity of the tube has been impaired. Dr. McWeeney,[1] of Dublin, has just recorded a case in which this accident occurred, and was apparently caused by the presence of an inflammatory infiltration of the walls of the gullet, a condition of phlegmonous œsophagitis. The patient was a man aged 40, who was admitted to the Mater Misericordiæ Hospital at one p.m. on November 18th, 1899, complaining of pain, not very intense, in the lower part of the chest, and of swelling in the neck. His face was deeply cyanosed, he seemed to be gasping for breath, the pulse was quick and weak, and his general condition was somewhat collapsed. The neck and face were much swollen on both sides, and palpation at once revealed that the swelling was due to subcutaneous emphysema. No treatment had any effect, the dyspnœa became more intense, and the emphysema crept up to the eyelids and down to the thorax, death occurring at eight p.m. He was an alcoholic person, who used to vomit every morning, and was much given to "dry retching." On the evening before his death he had tinned salmon for dinner and the following morning had retched worse than usual. Nevertheless he went to his work, where he noticed that his neck was swollen; his breathing then became difficult, he began to have pains in the chest, and he left his work about noon. A careful account is given by Dr. McWeeney of the post-mortem appearances found in this case, and he tabulates a number of other cases the records of which he has been able to trace, and the conclusion at which he arrives from a careful study of existing data is that the two main factors which are operative in causing rupture of the macroscopically normal gullet are (*a*) softening of the coats, and (*b*) sudden increase of pressure from within. The softening is due partly to intra-vital digestion, and partly (at least in the case which he observed) to inflammatory infiltration. The intra-vital digestion is to be accounted for by circulatory disturbance, which in his case took the form of venous thrombosis, and to prolonged sojourn of peptic matters in the gullet from prolonged retching. The increased pressure from within is doubtless ascribable, he says, to want of co-ordination in the muscular action of the tube.

[1] Lancet, July 21.

